# Rib fractures with heamothorax after labor: a case report

**DOI:** 10.4076/1757-1626-2-8950

**Published:** 2009-09-10

**Authors:** Vasileios K Kouritas, Ioannis Baloyiannis, Nikolaos Desimonas, Alexandros Daponte, Maria Kouvaraki, Kostas Hatzitheofilou

**Affiliations:** 1 Deptartment of Surgery, Larissa University Hospital, Mezourlo 41 110, PO Box 1425, Greece; 2 Department of Cardiothoracic Surgery, Larissa University Hospital, Mezourlo 41 110, PO Box 1425, Greece; 3 Department of Obstetrics and Gynecology, Larissa University Hospital, Mezourlo 41 110, PO Box 1425, Greece

## Abstract

**Introduction:**

Maternal thoracic trauma during labor is extremely rare.

**Case presentation:**

A woman was presented at the Accident and Emergency Department complaining of pain over the lower thorax bilaterally which started after a difficult delivery when the obstetrician forced her lower thorax. Small right-sided haemothorax and rib fractures bilaterally were diagnosed and she was admitted to hospital. Her in-hospital stay and follow up was uneventful.

**Conclusion:**

Maneuvers during labor should be applied from trained personnel and should be performed safely.

## Introduction

It is known that a complicated delivery can be due to different reasons such as abnormal infant position, shoulder dystokia etc. In order to deliver the infant, the obstetrician or perinatal personnel may apply different kinds of maneuvers i.e. the fundal pressure in an effort to avoid the possibility of transforming the delivery to cesarean section [1].

Many complications result from these maneuvers. For example uterine rupture may occur after fundal pressure [2], while perineal lacerations and cephalo-heamatoma appear more frequently after such maneuvers application [3,4]. Scarce complications might also appear including the development of pleural effusion [5], or the spontaneous appearance of chylothorax [6]. One case of diaphragmatic rupture to due force application on a patient's abdomen during labor has also been reported [7].

We present an unusual case of bilateral thoracic trauma (rib fractures) and small right-sided heamothorax occurred by an attempted maneuver application during labor.

## Case presentation

**Figure 1:**
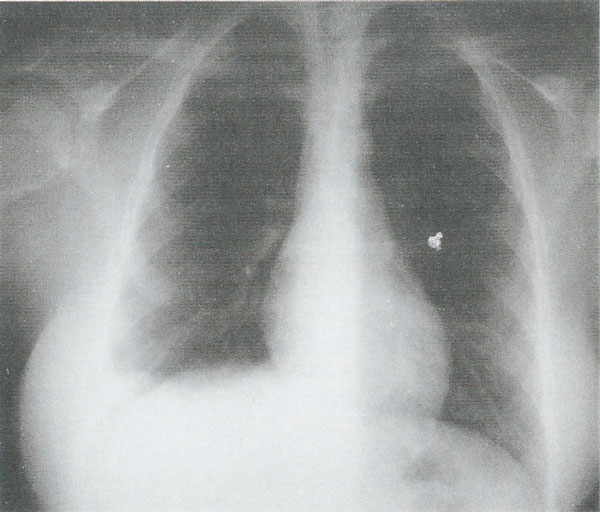
**Chest X-ray of the patient showing right-sided heamothorax**.

**Figure 2:**
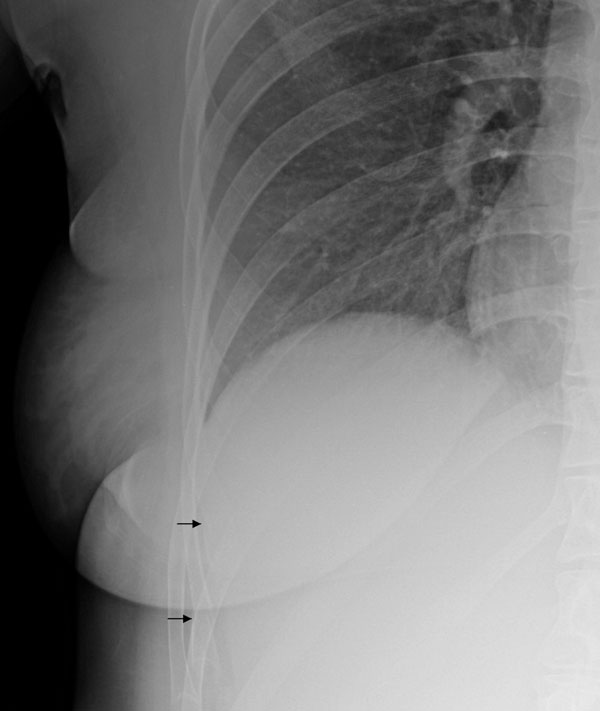
**Right rib cage X-ray of the patient showing fractures of the 8^th^ and 9^th^ rib**.

**Figure 3:**
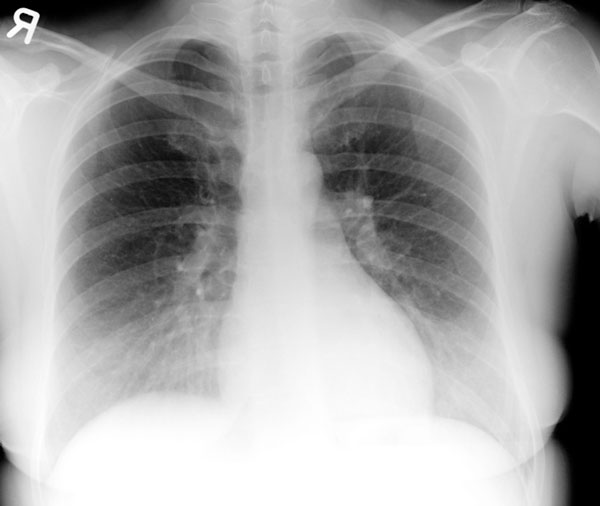
**Chest X-ray of the patient on follow up**.

A 29-year-old female (Greek Gipsy) was presented at the Accident and Emergency Department complaining of chest pain mainly located over the lower thoracic cage, bilaterally. The pain appeared immediately after delivery of her baby fifteen days ago and became progressively greater. The delivery was performed in a discrete, district hospital by obstetrics and gynecology medical personnel. In addition, the patient reported that "medical staff had to apply force on her chest in order to help delivery". The patient had a clean past medical history. On examination, tenderness above the previously mentioned area was verified. Tenderness over the right hypochondrium was also noted. Full blood count revealed anemia (Hematocrit 26.5%, hemoglobin 8.9 mg/dl). A possible pneumonic embolism was though to be the cause of the pain, but fibrinogen, D-Dimer values and arterial blood gases were normal. An electrocardiogram was performed and proven to be normal. Chest X-ray revealed a small right-sided haemothorax (Figure 1). Rib cage X-rays were also performed, that revealed 8^th^ and 9^th^ rib fractures of the right hemithorax (Figure 2) and 9^th^ to 11^th^ rib fractures of the left hemithorax. An abdominal ultrasound was finally performed which was also normal. The patient was admitted in hospital. She was discharged on painkillers two days later and referred to the outpatient department in for further follow up. The patient's pain subsided while the haemothorax was observed to resolve on her follow up chest x-ray fifteen days later (Figure 3).

## Discussion

In general, difficult deliveries due to a variety of reasons are described in the literature (i.e. shoulder dystokia, pelvic deformities, diabetic mother) [1]. The obstetrician and the perinatal personnel who faces a complicated delivery may apply a number of different maneuvers in order to achieve childbirth. Their use and efficiency is nowadays questioned. Fundal pressure can lead to complications that can even be life-threatening, as is the case in uterine rupture, while delivery can be adversely prolonged [2]-[4]. The benefit of this maneuver in the progression of the delivery is controversial [8]. However, expulsive force can be increased up to 86% if fundal pressure and valsalva maneuver are used simultaneously [9]. Moreover, fundal pressure seems to be associated with uterine prolapse [10], perineal lacerations [10], or even rupture of the unscarred uterus [11]. In general, the current trend leys with the avoidance of fundal pressure application [2]. Obstetric maneuvers seems to be correlated with increased incident of perineal lacerations, cephaloheamotoma and caput succedaneum [3], while their application in shoulder dystokia cases did not adverse maternal outcome [12]. In addition, the indecision upon the need of a cesarean section may also lead to adverse effects for either the mother or the child.

Unusual maternal complications, apart from the genital system, during labor have been previously published. Zimmermann reported a diaphragmatic rupture when the obstetrician applied force over the upper abdomen of a patient [7]. A case of chylothorax has also been reported; this was generated by the Valsalva maneuver which was done when the obstetrician asked the patient to "push". The patient eventually required thoracotomy, thoracic duct ligation and pleurodesis [6].

To our knowledge, thoracic trauma of the mother during delivery is unusual and never reported before although complications such as the development of a pleural effusion due to great intra-pleural forces acting during labor are possible [5]. The development of a chylothorax during delivery needed vigorous treatment that ended up in ligation of the thoracic duct, as mentioned before [6]. Rib fractures can be a serious traumatic finding which may be complicated by heamothorax or pneumothorax and which can lead to thoracocentesis or chest tube insertion. Of course such a complication is possible to surcharge negatively to the postpartum recovery of the mother and lead to possible prolonged in-hospital stay as well as further complications may arise as is possible after thoracic trauma.

Doctors or specialized perinatal personnel should always bear in mind possible complications arising from maneuvers made for the benefit of patients in critical conditions as is the case in complicated deliveries. In such cases, 'heroic' maneuvers, wrongly, might be applied. In general, many cases do not make it to publicity due to medical - legal implications of the perinatal team involved [13]. Despite the reluctance to publish such cases, anecdotal cases are published indicating the presence of the problem [13]. Experience and training is needed in order to perform any maneuvers correctly and safely, if not possible to do otherwise. In addition, a controlled environment when possible is needed, where help and/or operating theatre facilities can be sought. Classic examinations should be ordered after thorough identification of the traumatic mechanism. Finally, personnel dealing with deliveries should more diligently investigate postpartum complaints, in order to diagnose, treat and soothe the patient.

## Conclusion

In conclusion, medical interference in critical situations such as a complicated childbirth should be careful and concurrent with the good interest of both mother and infant. The benefit of maneuvers are under discussion, but always need to be applied with care (not with hard force), and knowing that there are possible complications, that must be excluded if the patient has pain or complaints.

## Competing interests

The authors declare that they have no competing interests.

## Consent

We were unable to gain consent for publication, but the following conditions are met: all reasonable attempts to gain consent have been made the patient is anonymous there is no reason to think that the patient or their family would object to publication.

## Authors' contributions

All authors had substantial contribution to patients management and treatment.

## References

[B1] Cunningham G Leveno KJ Bloom SL Hauth JC Gilstrap LC Wenstrom KD Williams Obstetrics 2005 McGraw-Hill

[B2] Wei SC Chen CP Uterine rupture due to traumatic assisted fundal pressure Taiwan J Obstet Gyneco 2006 45 170 172 10.1016/S1028-4559(09)60219-9 17197362

[B3] Garcia H Rubio-Espiritu J Islas-Rontrigez MT Risk factors for birth injuries Rev Invest Clin 2006 58 416 423 17408101

[B4] Cosner KR Use of fundal pressure during second-stage labor. A pilot study J Nurse Midwifery 1996 41 334 337 10.1016/0091-2182(96)00033-X 8828318

[B5] Gourgoulianis KI Karantanas AH Diminikou G Molyvdas PA Benign postpartum pleural effusion Eur Respir J 1995 8 1748 1750 10.1183/09031936.95.08101748 8586133

[B6] Cammarata SK Brush RE Hyzy RC Chylothorax after childbirth Chest 1991 99 1539 1540 10.1378/chest.99.6.1539 2036852

[B7] Zimmermann T An unusual trauma in labor: diaphragmatic rupture Zentralbl Gynakol 1999 121 92 94 10096176

[B8] Merhi ZO Awonuga AO The role of uterine fundal pressure in the management of the second stage of labor: a reappraisal Obstet Gynecol Surv 2005 60 599 603 10.1097/01.ogx.0000175804.68946.ac 16121114

[B9] Buhimschi CS Buhimschi IA Malinow AM Kopelman JN Weiner CP The effect of fundal pressure manoeuvre on intrauterine pressure in the second stage of labor BJOG 2002 109 520 526 10.1111/j.1471-0528.2002.01399.x 12066941

[B10] Tukur J Omale AO Abdullahi H Datti Z Uterine prolapse following fundal pressure in the first stage of labour: a case report Ann Afr Med 2007 6 194 196 10.4103/1596-3519.55697 18354946

[B11] Pan HS Huang LW Hwang JL Lee CY Tsai YL Cheng WC Uterine rupture in an unscarred uterus after application of fundal pressure. A case report J Reprod Med 2002 47 1044 1046 12516327

[B12] Mazouni C Menard JP Porcu G Cohen-Solal E Heckenroth H Gamerre M Bretelle F Maternal morbidity associated with obstetrical manoeuvres in shoulder dystocia Eur J Obstet Gynecol Reprod Biol 2006 129 15 18 10.1016/j.ejogrb.2005.11.006 16338049

[B13] Simpson KR Knox GE Fundal pressure during the second stage of labor MCN Am J Matern Child Nurs 2001 26 64 70 10.1097/00005721-200103000-00004 11265438

